# Biological Control of a Phytosanitary Pest (*Thaumatotibia leucotreta*): A Case Study

**DOI:** 10.3390/ijerph18031198

**Published:** 2021-01-29

**Authors:** Sean D. Moore

**Affiliations:** 1 Citrus Research International, P.O. Box 5095, Walmer, Port Elizabeth 6065, South Africa; seanmoore@cri.co.za; 2 Centre for Biological Control, Department of Zoology and Entomology, Rhodes University, P.O. Box 94, Makhanda 6140, South Africa

**Keywords:** false codling moth, citrus, systems approach, preharvest control, chemical residues

## Abstract

*Thaumatotibia leucotreta*, known as the false codling moth, is a pest of citrus and other crops in sub-Saharan Africa. As it is endemic to this region and as South Africa exports most of its citrus around the world, *T. leucotreta* has phytosanitary status for most markets. This means that there is zero tolerance for any infestation with live larvae in the market. Consequently, control measures prior to exporting must be exemplary. Certain markets require a standalone postharvest disinfestation treatment for *T. leucotreta*. However, the European Union accepts a systems approach, consisting of three measures and numerous components within these measures. Although effective preharvest control measures are important under all circumstances, they are most critical where a standalone postharvest disinfestation treatment is not applied, such as within a systems approach. Conventional wisdom may lead a belief that effective chemical control tools are imperative to achieve this end. However, we demonstrate that it is possible to effectively control *T. leucotreta* to a level acceptable for a phytosanitary market, using only biological control tools. This includes parasitoids, predators, microbial control, semiochemicals, and sterile insects. Simultaneously, on-farm and environmental safety is improved and compliance with the increasing stringency of chemical residue requirements imposed by markets is achieved.

## 1. Introduction

A phytosanitary or quarantine pest is a pest of potential economic importance to an area where it is not yet present [1]. One of the prospective threats to the area lies in the importation of fresh commodities that can host the phytosanitary organism, from its native region. As global trade of susceptible fresh commodities increases, so does the risk of spread of phytosanitary pests and diseases [2]. Air passenger baggage can be another source of spread of phytosanitary organisms, but it is unclear to what extent, particularly for Lepidoptera [3]. Problems with invasive species can be further exacerbated by changes in climate, habitats, soil nitrogen levels, and atmospheric carbon dioxide levels [4]. The economic costs associated with alien species have been estimated to amount to about 5% of the world gross national product (GNP), underscoring the importance of phytosanitary protection [5]. This value would be even higher if data for all countries, species, and processes existed [6].

Phytosanitary protection is achieved through regulatory plant protection, which aims to safeguard agricultural crops from such pests and diseases [7]. Such regulations and programmes must continually be in place to prevent pests from being transported along with these commodities [7]. To this end, the International Plant Protection Convention (IPPC) was initiated in 1952, as an international treaty to prevent the spread and introduction of pests of plants and plant products, and to promote measures for their control [7]. The IPPC espouses International Standards for Phytosanitary Measures (ISPMs) that are adopted by its more than 180 member countries. The IPPC definition of a phytosanitary measure includes legislation, regulations, or official procedures, including treatments, to prevent the introduction and/or spread of quarantine pests [8]. A variety of methods are available to reduce the risk of invasive species accompanying commodities shipped across natural barriers to result in invasion. These are most often standalone phytosanitary treatments that result in an acceptably high reduction in risk of introduction of live organisms with the commodity [1]. A common example would be a cold disinfestation treatment that provides Probit 9 efficacy (99.9968% mortality) [9]. However, treatments may occasionally be more complex, such as nonhost status [10], pest-free areas [11] or a systems approach [12].

*Thaumatotibia leucotreta* (Meyrick) (Lepidoptera: Tortricidae), known as the false codling moth, is one such phytosanitary organism [13]. It is a pest of citrus, peppers, pomegranates, peaches, and other crops in sub-Saharan Africa [14]. It is endemic to this world region and, consequently, it has phytosanitary status for most markets to which these fresh products are exported [15]. This includes Europe, the United States of America (USA), and many countries in the Far East. This status equates to a zero tolerance for the presence of the pest in the market. This is a situation that South Africa takes very seriously, as just the southern African citrus industry alone (South Africa, Zimbabwe, and Eswatini) exports around 2.1 million tons of fruit per annum, at a value of around 1.3 billion United States dollars (USD) [16]. Consequently, repercussions for inadequate compliance could be dire.

It is, therefore, imperative that *T. leucotreta* is controlled to as close to a nondetectable level as possible in the country of origin, before fruit are exported. All export markets that regulate *T. leucotreta* as a phytosanitary pest require a quarantine treatment of some sort [15]. For most markets, this is in the form of a standalone cold disinfestation treatment. However, for Europe (which together with the United Kingdom (UK) takes more than 40% of South Africa’s export citrus), a systems approach is accepted as an effective alternative to a standalone disinfestation treatment [13,17]. Cold disinfestation of the large volumes of fruit exported to Europe would be impossible, not only due to logistical and infrastructural challenges, but also, more importantly, due to the fact that certain citrus types and cultivars are susceptible to chilling injury [18,19] and are not suitable for export under such cold treatment conditions.

A systems approach is defined as “the integration of different risk management measures, at least two of which act independently, and which cumulatively achieve the appropriate level of protection against regulated pests” [8]. Aluja and Mangan [20] define a systems approach as “the integration of pre and postharvest practices, from the production of a commodity to its distribution and end use, that cumulatively meet predetermined requirements for quarantine security”. The systems approach for *T. leucotreta* consists of three measures: (1) preharvest controls and measurements and post-picking sampling, inspection, and packinghouse procedures, (2) post-packing sampling and inspection, and (3) shipping conditions. All in all, Moore et al. [17] and Hattingh et al. [13] listed 13 different components within these three different measures of the systems approach. Each component serves as an important risk filter, and they cumulatively reduce the overall risk of a larva surviving in a consignment of fruit to less than that associated with the efficacy standard often used for a standalone phytosanitary treatment, being Probit 9 [13,17], i.e., no survivors when treating a population of 93,613 insects [1,21,22].

Although effective preharvest control measures for phytosanitary pests are important under all circumstances, they are most critical where a standalone postharvest disinfestation treatment, which would provide adequate postharvest risk reduction on its own, is not applied. However, in order for a systems approach to succeed, every single measure and step in the system must be optimally applied; otherwise, inadequate overall risk reduction may result. Consequently, preharvest control measures are an extremely important component of the systems approach and, thus, need to be highly effective. Conventional wisdom may lead a belief that effective chemical control tools are, thus, imperative, generally being considered more effective and faster acting than their biological alternatives, particularly considering the zero-tolerance status of *T. leucotreta* for export markets. The downside of employing this rationale is that, although chemical pesticide residues spell no risk to consumers, due to strict compliance with scientifically determined maximum residue levels [23], there is evidence that their injudicious use will result in greater health risks to the user [24] and the environment [25]. Furthermore, the South African government has legislated the elimination or restriction of several insecticides since the late 1970s, fueling the need for safer alternatives and underscoring the intentions of the government to establish the so-called “South African National Bio-Economy Strategy” [26].

Confounding the belief that chemical pesticide tools may be unavoidable for control of a phytosanitary pest, for which there is no tolerance, we demonstrate here that it is possible to effectively control *T. leucotreta* to a level acceptable for a phytosanitary market, using only biological control tools, considering the further risk reduction added by the postharvest measures applied thereafter. Simultaneously, compliance with the increasing stringency of chemical residue requirements imposed by markets, from a regulatory perspective and even more stringently through arbitrary retailer standards, is achieved [27]. Consequently, even within the strict paradigms of organic production of fruit, the phytosanitary risk of *T. leucotreta* for export markets can be sufficiently mitigated.

Preharvest control of *T. leucotreta* is considered to consist of three tiers. The foundation is orchard sanitation, without which no control programme can be effective. Overlaid onto this is an areawide control technique, such as the sterile insect technique or mating disruption. Finally, orchard specific control measures are applied, such as parasitoid releases or insecticidal sprays. Preharvest monitoring is conducted using pheromone traps and by evaluating fruit infestation from designated data trees [17]. However, as *T. leucotreta* is now primarily a phytosanitary pest, rather than an economic pest, these monitoring tools are no longer used to determine whether intervention is warranted or not, i.e., threshold purposes [28]. This is to say that the economic losses that the pest could cause pale into insignificance with the prospect of a live larva being detected in the marketplace, for which there is zero tolerance. Consequently, contrary to their original purpose, traps are now used as a precision management tool, such as for optimizing timing of treatments [28]. Fruit infestation monitoring is used to determine whether infestation is adequately low for the orchard to be suitable for export to a market for which *T. leucotreta* is a regulated phytosanitary organism [13].

Orchard sanitation involves the regular collection and removal of infested and injured fruit from the orchard, both hanging and fallen fruit, as well as the destruction of this fruit outside of the orchard. Moore and Kirkman [29] demonstrated that weekly orchard sanitation conducted from December to June could reduce fruit infestation by an average of 75%.

Here, we provide the details and efficacy of specific biological control measures, be they areawide technologies or orchard-targeted technologies, that are overlaid onto the foundation of orchard sanitation. However, before we do so, it is important that we clarify what we define as biological control. Biological control has been defined as the introduction and manipulation of natural enemies by man to control pests [30], or the use of a population of one organism to reduce the population of another organism [31]. Along with parasitoids, predators, and pathogens, the second definition implies inclusion of a technology such as the sterile insect technique (SIT). We choose to expand this definition to also include derivatives of biological organisms, specifically semiochemicals, as asserted by Mweresa et al. [32].

## 2. Sterile Insect Technique

The IPPC categorizes sterile insects as beneficial organisms [33]. Consequently, SIT can be considered a form of biological control. Furthermore, the IPPC defines SIT as a method of pest control using areawide inundative releases of sterile insects to reduce fertility of a field population of the same species [8]. The concept of SIT is that mass-reared males, sterilized through irradiation, mate with fertile wild females, which then lay only infertile eggs [34,35]. An important principle for success with SIT is achieving a sufficient “overflooding ratio” of sterile males to wild females [34]. The released sterile males must adequately outnumber the wild males in the release area to introduce sufficient sterility into the wild population to overcome the natural rate of increase of the wild females [36]. This overflooding ratio varies from insect to insect [34]; however, for *T. leucotreta*, it has been determined to be a minimum of 10 sterile to one wild male [37]. If other on-farm practices, such as orchard sanitation, are good [29], the size of successive generations of the pest is, thus, reduced [36]. 

SIT for *T. leucotreta* has been commercially implemented in the citrus and table grape industries in South Africa since 2007 [38]. It is now applied in over 18,000 ha in several different regions within three provinces throughout the country [39].

SIT was commercialized after a successful pilot trial in citrus. As a standalone treatment in a semicommercial trial, SIT reduced *T. leucotreta* infestation in 35 ha of Washington Navel orange orchards by 95.2%, relative to an untreated control orchard [38]. These findings led to commercial implementation for control of *T. leucotreta* within an integrated programme, initially in the Western Cape Province (since 2007) and more recently in various other production regions, and it is proving extremely effective [40], having reduced moth catches by 99%, fruit infestation by 96%, and export rejections by 89% since the inception of the programme [36]. Consequently, SIT is strongly recommended as a foundational areawide technology in regions of the country where it is commercially available. Furthermore, apart from being highly effective, it is easily implementable for farmers, as the moths are released and the efficacy of the programme is monitored by the service provider, thus relieving the farmer to focus on other associated good management practices.

## 3. Mating Disruption

Mating disruption is the broadcasting of sex attractant pheromones within a crop so as to disrupt reproduction of insect pests and, thus, suppress population build up [41]. Most often, this is the female sex pheromone, which disrupts males from mating. Once the pheromone is delivered, the behaviour of males is changed to achieve mating disruption by one of two ways: (1) by competitive attraction where males are diverted from orienting to females due to competing attraction of nearby false trails emanating from pheromone dispensers, or (2) by noncompetitive means, where exposure to synthetic pheromone subsequently reduces or blocks the male’s ability to sense pheromone normally [42,43]. The latter could be achieved by negating the male’s ability to respond to pheromone or by camouflaging the location of a pheromone-emitting female.

Mating disruption for *T. leucotreta* was introduced into South Africa in the 1990s. Products were initially based on pheromone from the oriental fruit moth, *Grapholita molesta* (Busck), which showed some efficacy against *T. leucotreta*, but insufficient to warrant commercialization of the products [44]. Subsequently, trials were conducted with the product Isomate (Shin-Etsu, Japan), using the *T. leucotreta* pheromone, in Navel orange orchards. Isomate, reduced *T. leucotreta* infestation of fruit from December to the end of April by 55% in an orchard considered to have high pest abundance, and by 75% in an orchard considered to have low pest pressure [45]. More importantly, these reductions were 86% and 95%, respectively, in later evaluations shortly before harvest. In another trial, it was found that, by initiating mating disruption in October, as opposed to November, efficacy was improved from a 51% to 80% reduction in infestation [46]. 

Steyn [47] showed that *T. leucotreta*’s disruption profile closely follows the predictions of the hybrid disruption profile of Miller and Gut [41], suggesting that *T. leucotreta* is likely disrupted competitively at low doses and noncompetitively at high doses. However, the pheromone dose required to induce this shift from competitive to noncompetitive disruption was 192 g per ha [47]. Unfortunately, release rates were not reported. However, this dose is higher than any of the recommended registered doses of the available mating disruption products [48]; thus, in practice, mating disruption of *T. leucotreta* remains competitive in nature and, therefore, most effective when the pest population is at a low level [41].

Four registered products are available for *T. leucotreta* control: Isomate FCM, Checkmate FCM-F (Suttera, Bend, OR, USA), Splat-FCM (ISCA Technologies, Riverside, CA, USA), and X-Mate (Insect Science, Tzaneen, South Africa) [46,48,49]. Isomate consists of thin polyethylene tube dispensers containing liquid sex pheromone that is released through the tube walls into the atmosphere. Checkmate is a pheromone-containing capsule suspension formulation. Splat FCM is an amorphous polymer matrix, containing pheromone. X-Mate consists of cellulose disc dispensers containing liquid sex pheromone. In regions of the country where SIT is not commercially available, mating disruption is recommended as the areawide technology of choice. Its efficacy is generally similar to that of SIT, but it requires more labour input from the grower, particularly where high-density deployment of dispensers is required, such as in the case of Isomate. Splat-FCM is applied for the farmer by the service provider, due to the specialized application equipment required.

## 4. Attract-and-Kill

Attract-and-kill technology is similar to mating disruption in that it also makes use of semiochemicals for achieving the end of controlling the pest. However, in attract-and-kill, semiochemicals are used in combination with insecticides (most often), sterilants, or insect pathogens [50]. The insect responding to the semiochemical lure will encounter the insecticide or sterilizing agent, which effectively eliminates it from the population after a short time [51].

Currently, only one product based on the attract-and-kill technology is commercially available for management of *T. leucotreta*. The product, under the name Last Call FCM (Insect Science, Tzaneen, South Africa), consists of a blend of synthesized female sex pheromone and a pyrethroid, in the form of a gel. The combination attracts and kills male moths [46]. As with mating disruption, the technique is density-dependent, but it does not appear to be as effective as the currently available mating disruption products. Attract-and-kill technology should, therefore, not be used as the sole control method, unless the FCM population is at a low level [46].

## 5. Parasitoids

*Thaumatotibia leucotreta* has a range of natural enemies that suppress it in the field [14]. The most effective biological control agent against *T. leucotreta* is the egg parasitoid, *Trichogrammatoidea cryptophlebiae* Nagaraja (Hymenoptera: Trichogrammatidae) [52,53]. Where undisrupted, parasitism from naturally occurring *T. cryptophlebiae* reached between 80% and 100%, causing from a 67% reduction in *T. leucotreta* infestation in Navel oranges from December to harvest to total elimination of detectable *T. leucotreta* infestation by harvest [46]. However, due to a number of abiotic factors, the effectiveness of *T. cryptophlebiae* in controlling *T. leucotreta* can be variable [54]. Additionally, it is highly sensitive to chemical pesticides, and injudicious selection of treatments for other pests may effectively reduce the parasitoid to negligible levels [46,55].

Initial studies on the effectiveness of *T. cryptophlebiae* augmentation were conducted during the 1970s and 1980s [56,57,58]. Schwartz [57] demonstrated up to a 75% reduction in *T. leucotreta* larval infestation of fruit, by releasing more than 862,000 parasitoids per hectare over a 3-month period. Newton and Odendaal [59] released 1.5–3.8 million parasitoids per hectare, divided into weekly releases. Results were variable, but larval populations were reduced by up to 61%. However, such high density and frequent releases as used in both of these studies are likely to be impractical and expensive if conducted on a commercial scale. Admittedly, Newton [58] and Newton and Odendaal [59] referred to their strategy as inundative. More recently, it was shown that a more practical and affordable inoculative release approach can be effective [49]. As few as 100,000 parasitoids released per hectare (25,000 parasitoids per month for four consecutive months) reduced *T. leucotreta* infestation by up to 60% [49]. Imperative to achieving this level of success is the early initiation of releases. Releases initiated in October (spring) resulted in a greater reduction in *T. leucotreta* infestation than those initiated during November and December [60].

Historically, commercial mass rearing and augmentation of *T. cryptophlebiae* have been conducted by Cederberg Insectary in Citrusdal in the Western Cape Province [61] and at Zebediela Estate in Limpopo Province [62,63]. Currently, there is only one commercial insectary doing this: Vital Bugs in Limpopo Province. Consequently, augmentation of *T. cryptophlebiae* can only be conducted over a fairly limited area; thus, conservation of the naturally occurring population remains an attractive, practical, and universally available option. If parasitoids are available for augmentation, release is easily conducted by growers in the form of parasitized eggs placed at various well-spaced intervals throughout orchards, on a monthly basis for 4 months; thus, it is not an onerous process. However, if this cannot be initiated early in the season (spring or early summer), possibly due to the detrimental impact of chemical sprays applied against other pests, then augmentation should not be considered a viable option. 

Several other parasitoids of *T. leucotreta* have been recorded, as listed by Newton [62] and Moore [53]. Probably the most effective of these is the larval parasitoid, *Agathis bishopi* (Nixon) [64,65,66]. However, no mass rearing or augmentation programme exists for this or any of the other species. Consequently, the only benefit that can be derived from them is through conservation, by following a biointensive integrated pest management (IPM) approach.

## 6. Predators

Ants are considered as important pests in citrus trees. The main problematic species are the pugnacious ant, *Anoplolepis custodiens* (Smith), and the brown house ant, *Pheidole megacephala* (Fabricius) [67], which do not cause direct damage to citrus plants but will defend honeydew-producing pests on citrus from their natural enemies and even transport their immature life stages to new foliage [14,68]. This includes pests such as citrus mealybug, *Planococcus citri* (Risso), soft scales (*Coccus hesperidum* L. and *Pulvinaria aethiopica* (De Lotto)), and waxy scales (*Ceroplastes brevicauda* Hall and *C. destructor* Newst.) [14,68]. Furthermore, the ants can disrupt the natural enemies of other pests, such as California red scale, *Aonidiella aurantii* (Mask.), causing outbreaks of these pests [69].

This emphasizes the importance of keeping ants out of citrus trees. However, on the orchard floor, ants can act as an important and effective predator of arboreal pests that pupate in the soil, such as fruit flies, *Ceratitis* spp. (Wied.), bollworm, *Helicoverpa armigera* (Hüb), and *T. leucotreta* [70]. Bownes et al. [70] planted pupae of these three species in a citrus orchard in plots where *A. custodiens* and *P. megacephala* had been bait-poisoned and in control plots. Pest survival was significantly lower for all three species in control plots, indicating significant predation by ants. Predation on *T. leucotreta* pupae was significantly higher than for the other two species. Consequently, the practice of barrier banding citrus trees to exclude ants, as opposed to poisoning, is strongly recommended to growers [14,71]. This is a labour-intensive process, including not only the deployment of the bands, but also their regular maintenance, and it would probably not be justified by a grower unless they are truly committed to an IPM philosophy.

Other predators that have been recorded include the hemipteran egg predators, *Orius* sp. and *Orius insiduosus* [62,72], the larval predator, *Rhynocorus albopunctatus* (Stål), and the egg/larval mite predator, *Pediculoides* sp. [72]. Shrews have also been implicated as predators of pupae [73]. Obviously, these generalist predators cannot be directly exploited for their benefit; however, as with ants, pursuance of an IPM programme and allowing grasses and herbs to grow between the rows of trees would lead to greater habitat complexity, increased niche diversity, and the development of a richer predator community [74].

## 7. Granulovirus

The Cryptophlebia leucotreta granulovirus (CrleGV) was first isolated from the Ivory Coast from infected *T. leucotreta* larvae [75]. Although the genus of the host has changed from *Cryptophlebia* to *Thaumatotibia* [76], the original name of the virus has remained unchanged [77]. Other isolates were subsequently identified, including the Cape Verde isolate (CrleGV-CV3) and several South African isolates (i.e., CrleGV-SA) [78,79,80]. The development of CrleGV as a biopesticide in South Africa began in 1998, leading to its evaluation in several field trials from 2000 [53,81]. Two different South African isolates have since been formulated and registered as the commercial products Cryptogran (River Bioscience, South Africa), Cryptex, and Gratham (both Andermatt Biocontrol, Switzerland) for field application [27]. These are registered at rates equivalent to 5 × 10^12^ occlusion bodies (OBs)/ha in the case of Cryptogran and 6.6 × 10^11^ OBs/ha in the case of the other two products.

CrleGV has been extensively tested in the field for many years with more than 50 trials completed, and it has been used commercially over tens of thousands of hectares annually for almost two decades [27,77,81]. Trial results show that applications of CrleGV have consistently led to significant reductions in fruit infestation in relation to untreated controls. The highest reduction in infestation was reported to be 92%, while the lowest was 27% [77]. While such variability in levels of suppression may be undesirable, this underscores the importance of an IPM programme consisting of several technologies to cumulatively reduce pest levels. Several of the trials also showed that the addition of an adjuvant such as molasses often significantly improved the efficacy of the treatment, frequently achieving levels of control similar to the chemical alternatives and, in some cases, outperforming certain chemicals [77]. In one case, this was attributed to possible resistance in the *T. leucotreta* population [82]; however, in others, it was simply superior efficacy.

The granulovirus products are probably the most widely used of all products for *T. leucotreta* control, including the chemical insecticides, bearing testimony to not only their efficacy, but also their affordability and ease of application. The products are applied using conventional pesticide spray equipment and can be tank-mixed with other pesticides without risk of compromised efficacy.

Despite field performance generally being good, further improvement of field efficacy is being sought, through combining isolates of the same virus. As discussed by López-Ferber et al. [83], the use of pure genotypes or low-genetic-diversity isolates in products, would be less effective than products containing virus with greater genetic heterogenicity. Preliminary field trials, combining two regionally distinct CrleGV isolates, have already been reported to yield better results than those achieved with the standard single isolate CrleGV product [84].

Probably the most important shortcoming of baculoviruses is their sensitivity to ultraviolet (UV) degradation and, hence, rapid breakdown in the presence of direct sunlight [85,86,87]. Consequently, another means by which improvement in the efficacy of CrleGV is being sought is through selection for UV resistance. By exposing virus samples to conditions simulating normal daylight UV, propagating these in larvae and re-exposing them to UV, after five cycles, LC_50_ (the concentration required to kill 50% of sampled individuals in a population) and LC_90_ (the concentration required to kill 90% of sampled individuals in a population) were reduced 1226- and 563-fold [88,89]. This suggests successful isolation of UV-resistant virus, confirmed by associated genetic changes identified in 14 single-nucleotide polymorphisms (SNPs) [88]. The genetic stability of this selection and its efficacy in the field must still be confirmed.

## 8. Nucleopolyhedrovirus

Recently, the first nucleopolyhedrovirus (NPV) in the Grapholitini tribe of Tortricidae was discovered [90], being the NPV of the litchi moth, *Cryptophlebia peltastica* (Meyrick), an African pest species [91]. The Cryptophlebia peltastica NPV (CrpeNPV) was isolated and characterized genetically and biologically, demonstrating not only that it is a unique virus [90], but also that its virulence extends beyond its homologous host. In laboratory bioassays, CrpeNPV was found to be similarly virulent to the codling moth, *Cydia pomonella* (L.), and *T. leucotreta* as to *C. peltastica* and marginally more virulent than the homologous GVs of these species [92,93].

Preliminary field trials have already been conducted with CrpeNPV against *T. leucotreta* in citrus at rates of between 5 × 10^11^ and 5 × 10^13^ OBs/ha [94]. Very promising results were recorded, with infestation being reduced by over 90% with a single application in one case [94].

CrpeNPV has also been evaluated for use in combination with CrleGV, with some mixtures identified to have a synergistic effect, resulting in an increase in insecticidal activity [95,96]. However, additional research is still needed. The commercial formulation of CrpeNPV, either on its own or in combination with another virus such as CrleGV, could prove invaluable to future IPM programmes for *T. leucotreta*, not only on citrus, but also even more so on crops where more than one grapholitinid is a pest, such as on macadamias, where *T. leucotreta*, *C. peltastica*, and *T. batrachopa* (Meyrick) can be a problem.

The appearance of a CrpeNPV-based product on the market is imminent. It can be expected that its mode and ease of application and its affordability will be as for the granulovirus products.

## 9. Entomopathogenic Fungi

Laboratory bioassays against final instar *T. leucotreta* identified three entomopathogenic fungal (EPF) isolates with the greatest pesticidal potential, two *Metarhizium anisopliae* (Metchnikoff) Sorokin (Hypocreales: Clavicipitaceae) isolates and one *Beauveria bassiana* (Balsamo) Vuillemin (Hypocreales: Cordycipitaceae) isolate [97,98]. All three isolates were originally identified from soil samples collected from citrus orchards and surrounding refugia within citrus growing regions of the Eastern Cape Province of South Africa [99]. Results of sand-conidial bioassays indicated that fungal-induced mycosis of *T. leucotreta* of greater than 80% was achievable when applied at a rate of 1 × 10^6^ conidia/g soil with average LC_50_ values of 6.26 × 10^5^, 1.92 × 10^6^, and 1.98 × 10^5^ conidia/mL for the selected *M. anisopliae* and *B. bassiana* isolates [97,98]. These isolates also showed good persistence under semi-field conditions. All three isolates were recovered from within the upper 5 cm soil surface over 6 months after application in the field [100].

Thereafter, large-scale field trials were conducted with application of the three selected isolates to the soil surface underneath the canopy of citrus trees at rates ranging from 1 × 10^12^ to 1 × 10^14^ spores/ha, using a commercial tractor-drawn spray machine [101]. Weekly fruit drop surveys were conducted to determine the mean *T. leucotreta*-infested fruit per tree per week and any percentage reduction in *T. leucotreta* infestation that resulted from fungal application. In comparison to the control treatments, all three isolates used were capable of reducing *T. leucotreta* infestation in the field. The *B. bassiana* isolate performed best, with an average reduction in *T. leucotreta* infestation of 82%, measured over a 32-week period after a single application in spring. Over time, the reduction in *T. leucotreta* infestation remained constant for the *B. bassiana* isolate throughout the trial period, but declined for both *M. anisopliae* isolates (54.01% to 35.02%) and (47.39% to 27.65%) from 10 weeks after treatment to 32 weeks after treatment [101].

Commercial development of these isolates for application against the soil-dwelling life stages of *T. leucotreta* is underway. In the meantime, two other isolates of *B. bassiana* are already registered for use against *T. leucotreta* on citrus [102]. However, this is as a foliar application, targeted against the eggs and neonate larvae. Efficacy with this mode of application is not always satisfactory, due mainly to the UV sensitivity of the fungi and, hence, the short residual activity [102]. Although fairly extensive general use is made of these existing EPF products, their specific use against *T. leucotreta* is not commonplace, due largely to their limited efficacy. Consequently, development of an effective formulation to overcome UV sensitivity and other environmental hurdles is desirable in order to achieve more consistent efficacy with foliar application. In the meantime, efficacy can be optimized through accurate timing of applications with peaks in egg laying and frequent reapplication [103].

It is expected that, once the soil-targeted formulations become available, the use of EPF against *T. leucotreta* will increase, as not only will they be more effective, but application through the irrigation (microsprinkler) system will also be even easier than conventional spraying.

## 10. Entomopathogenic Nematodes

Malan et al. [104] and Steyn et al. [105] demonstrated in laboratory bioassays that several locally collected species of entomopathogenic nematodes (EPNs) were all highly virulent to *T. leucotreta* larvae at rates as low as 20 infective juveniles (IJs)/cm^2^. These were *Steinernema* sp., *Heterorhabditis bacteriophora*, *S. yirgalemense*, *S. khoisanae*, *H. zealandica*, *Heterorhabditis* sp., *Heterorhabditis baujardi*, and *Steinernema litchii*. These results provided strong evidence of noteworthy potential of the EPNs for the control of *T. leucotreta* in the field. Consequently, Malan and Moore [106] conducted small-scale field trials to examine infection of sentinel *T. leucotreta* larvae with EPNs in a citrus orchard environment. *Heterorhabditis bacteriophora*, *H. zealandica*, and *S. khoisanae* were applied at concentrations ranging from 5 to 80 IJs/cm^2^. The *Heterorhabditis* species were the most effective, with up to 90% infection of *T. leucotreta* larvae and detectable persistence for 49 days, albeit at a reduced level. 

These results were confirmed with significant efficacy recorded with *S. yirgalemense* and *S. jeffreyense* against *T. leucotreta* in semi-field trials in other crops (vineyards, avocado, litchi, and macadamia), using a similar methodology [47,107].

Consequently, large commercial-scale field trials were conducted in citrus orchards in the Eastern Cape, Western Cape, and Mpumalanga provinces of South Africa from 2011 to 2013, geared toward directly testing the ability of EPNs to achieve pest reduction and crop protection in the orchards [108]. *Heterorhabditis bacteriophora* and *S. feltiae* were applied underneath tree canopies in large orchards, using tractor-drawn spray machinery or through the irrigation system. Successful control was recorded in several trials, with EPN infection of larvae frequently exceeding 80% and larval infestation of fruit being reduced by up to 88%, with persistence recorded for up to 5 months [108]. However, results were sometimes disappointing, with the main cause being suboptimal soil moisture, a factor that can be managed.

Furthermore, naturally occurring EPNs (*H. zealandica*) were shown to reduce fruit infestation by *T. leucotreta* by 59% in orchards where they were not suppressed by the nematicide, cadusafos, emphasizing the importance of EPN conservation in orchards [109].

As a result of these positive results, an *H. bacteriophora*-based product (Cryptonem) (River Bioscience, South Africa) was registered for use against *T. leucotreta* in citrus [102]. This registration was unfortunately later suspended, due to unfounded regulatory concerns about nontarget effects [110,111]. However, the registration was recently revived, and registration of a new *S. feltiae*-based product has also been approved. This is an extremely positive development, as it now provides registered and commercially available biological products for targeting of the previously ignored soil-borne life stage of *T. leucotreta* [108].

It remains to be seen how extensively these products are used once they become commercially available. Their mode of application will be through the irrigation system as described for the EPF products and, thus, very user-friendly. However, efficacy, perception of efficacy, and cost will also affect use. Generally, our trials have shown more consistent efficacy with EPF than EPN against the soil-dwelling life stages of *T. leucotreta*.

## 11. Discussion

Several different biological control technologies are available or under development for use against the phytosanitary pest, *T. leucotreta* on citrus in southern Africa (Table 1). These include the sterile insect technique, mating disruption, attract-and-kill, parasitoids for augmentation, baculoviruses, entomopathogenic fungi, and entomopathogenic nematodes. There is a total of 13 such products currently available with more that are imminent. This outnumbers the registered and available chemical active ingredients for management of this pest, i.e., spinetoram, chlorantraniliprole, triflumuron, teflubenzuron, fenpropathrin, cypermethrin, methoxyfenozide, and emamectin benzoate [46,49]. Additionally, the insect growth regulators (IGRs), triflumuron and teflubenzuron, have limited usefulness due to stringent residue restrictions from most export markets and the development of resistance by *T. leucotreta* [82]. The use of the pyrethroids, fenpropathrin and cypermethrin, is undesirable due to their extreme harmfulness to nontarget organisms [55], including the exceptionally effective *T. leucotreta* egg parasitoid, *T. cryptophlebiae* [46,59]. This leaves only the newer-generation products, namely, spinetoram, chlorantraniliprole, methoxyfenozide, and emamectin benzoate. Although these products can be highly effective (e.g., [77]), they are expensive and their use shortly before harvest may lead to residue problems in the market.

There is a general perception that biopesticides may not be as effective as their chemical alternatives [27]. However, Moore et al. [77] have shown in many cases that the granulovirus, CrleGV, for example, can be as effective as, or even more effective, than its chemical counterparts for *T. leucotreta* control. Similarly, another virus, the nucleopolyhedrovirus, CrpeNPV, was able to reduce *T. leucotreta* infestation to a comparable extent, i.e., by up to 90% [94]. SIT has been shown to reduce *T. leucotreta* fruit infestation by 95% in a single trial [38] and by 96% in an entire treated region of several thousand hectares over time [36]. Chemical control has rarely been reported to achieve this level of efficacy and certainly does not have the sustainability of a biological approach. Mating disruption has been reported to reduce *T. leucotreta* by up to 95% shortly before harvest [45,46]. *Thaumatotibia leucotreta* infestation was shown to be reduced by up to 75% through augmentation of an egg parasitoid [57], by 82% with the application of EPF to the orchard soil [101], and by 88% using EPNs [108].

These are obviously best-case scenarios, and efficacy with biologicals does tend to be more variable than with chemicals [27,112]. However, even if the efficacy of each intervention is well below the maximum potential efficacy of the treatment, *T. leucotreta* control has become far more sophisticated than was previously the case, more specifically entailing the combination of various control measures as opposed to relying on a single treatment option [46]. The levels of control achieved should, therefore, be seen as the sum of the efficacy of all the measures used in combination or in sequence. For example, if a grower practices orchard sanitation, applies EPNs to the soil, applies two foliar virus sprays, and uses mating disruption (i.e., five different treatments), the level of control may conservatively be 50%, on top of 50%, on top of 50%, and so on. Whereas each treatment in isolation may not be considered adequate where the population level is high or when producing for a phytosanitarily sensitive market, the combination would provide a satisfactory cumulative control of approximately 97% [46]. Furthermore, withholding from chemical intervention will effectively conserve natural enemies resident in the orchard, thus naturally augmenting this level of control.

Consequently, the standard *T. leucotreta* control programme recommended for citrus in South Africa is a foundation of regular diligent orchard sanitation, overlaid with an areawide control technology, such as SIT or mating disruption, and sprays (preferably virus products) applied at appropriate intervals, usually coinciding with peaks in egg hatch, forecast by moth flight peaks [49]. Control measures for *T. leucotreta* for the following season should be initiated immediately on completion of harvesting of the previous season, by collecting, removing, and destroying all fruit remaining in the orchard, both those hanging and those dropped to the ground [29]. This will remove not only all potentially infested fruit remaining in the orchard, but also the bridge for transfer of the *T. leucotreta* population from one season’s crop to the next, particularly if there is a period of at least 3 months between harvest and fruit set, the maximum development period for the soil-dwelling life stages of *T. leucotreta* during winter [113]. The recommended areawide control measure must be initiated early in the season (spring), while the *T. leucotreta* population is still low, after suppression by winter conditions, and before population numbers can start to build. This is because all of these technologies are negatively density-dependent for optimal efficacy to be achieved [36,41]. Approaches such as release of egg parasitoids and application of EPF or EPN to the soil should also be initiated this early in the season for optimal efficacy [60,101,108]. Virus sprays can be applied at any time during the season, as they leave no detectable residues on fruit and have no undesirable nontarget effects [27]. However, as residual efficacy is detrimentally affected by direct exposure to UV radiation [85,86,87], application of sprays should ideally be timed with a peak in *T. leucotreta* egg hatch, which can be fairly accurately estimated through monitoring of male adult activity in pheromone traps [28]. Furthermore, as *T. leucotreta* is multivoltine (at least six generations per year in southern African conditions) [62] and does not diapause [114], generations are not distinctly separated from one another. As the season progresses, the overlap of generations tends to increase and it becomes more difficult to distinguish generational peaks. Consequently, detection of these generational peaks and, thus, ideal timing of a virus spray is easier during the first half of the season, after the synchronizing effect of winter.

As *T. leucotreta* is regulated as a phytosanitary pest by most export markets, meaning that there is zero tolerance for the pest, there are no thresholds dictating when control measures should be applied and when it is not considered necessary. Pheromone trap-based thresholds were recommended several years ago when *T. leucotreta* was primarily considered an economic pest, but this is no longer the case [28]. However, according to the *T. leucotreta* systems approach protocols, if a very low fruit infestation threshold is surpassed in fruit from the designated data trees in an orchard during the final 12 weeks before harvest, an additional corrective control measure must be applied, regardless of what control measures were previously applied [13]. If this low threshold is surpassed during the last 4 weeks before harvest, the fruit need to either be subjected to a more stringent postharvest handling protocol or be withdrawn from export to a sensitive market [13].

The full *T. leucotreta* management programme that a grower chooses will ultimately depend on several factors: the grower’s assessment of the severity of the problem, the sensitivity of the market to the presence of *T. leucotreta* in the fruit, the severity of the postharvest handling conditions available and selected, the value of the particular cultivar, and the anticipated prices in the market place. None of these biological methods of control are incompatible with one another, possibly, surprisingly, not even mating disruption and SIT. In fact, superior efficacy was recorded against the closely related apple pest, *C. pomonella*, by combining these technologies [115]. Consequently, any of these technologies can be used in combination and/or in sequence in order to achieve the best result.

Adoption of a judiciously constructed biological control programme will lead to a sufficiently suppressed level of *T. leucotreta* occurrence at harvest to be suitable for export of fruit to a market that regulates *T. leucotreta* as a phytosanitary organism and exercises zero tolerance for the occurrence of any live larvae in a consignment of fruit. This threshold tolerance at harvest (as opposed to zero tolerance) is acceptable, as the fruit will still be subjected to several further steps that together will reduce the occurrence of *T. leucotreta*-infested fruit to a negligible level [13,17]. This includes (1) postharvest fruit inspections for *T. leucotreta* infestation on delivery at the packinghouse, to determine subsequent handling requirements, (2) packinghouse grading of fruit on the packing line, involving the removal of any fruit appearing to be damaged or infested, (3) inspection of a 2% fruit sample per pallet of fruit packed for export and rejection of any pallet in which live *T. leucotreta* was detected, (4) prescription of shipping condition options for each export consignment according to compliance with preceding steps of the systems approach, and (5) official phytosanitary certification of compliant consignments.

Moore et al. [17] calculated the overall risk mitigation efficacy of the systems approach as the proportion of fruit that could be infested with *T. leucotreta* after application of the systems approach, determining that it was 6–38 times less than the proportion associated with the Probit 9 (*p* ≤ 3.2 × 10^−5^) standard for a standalone cold treatment, being three survivors in 100,000 at the 95% confidence level, proven by zero survivors in 93,613 insects, i.e., a 99.9968% mortality [1]. Hattingh et al. [13] later reported improvements to the systems approach for *T. leucotreta*, still concluding that the maximum potential proportion of fruit that may be infested with live *T. leucotreta* after application of the improved systems approach is no greater than the proportion of fruit that may be infested after application of a Probit 9 efficacy postharvest disinfestation treatment to fruit with a 2% pretreatment infestation. Additionally, they determined that the probability of a mating pair surviving the systems approach, as a more biologically meaningful alternative to the quantification of a mortality level such as the Probit 9 reference, was far lower. This reasoning would be strengthened by the fact that *T. leucotreta* is a poor invader [116,117], having only been established in two regions where it is not indigenous, i.e., the Western Cape of South Africa [40,118] and Israel [119]. This is despite large volumes of *T. leucotreta*-susceptible produce being exported throughout the world for more than 100 years [120].

Another assessment that must be made is that of the cost of a biological programme versus a conventional programme, as this too will influence a grower’s decision on what products to use. The cost of the biological products range from 62–309 USD per hectare per application or per programme with the product, whereas the cost of the chemical products ranges from 73–382 USD per hectare (Table 2). The areawide approaches, SIT, mating disruption, and attract-and-kill, are understandably expensive, as their efficacy spans several months, covering most of the growing season, as is also the case with parasitoid augmentation. However, the chemical sprays are on average a lot more expensive than the biological sprays. In comparing programmes, no programme is likely to be entirely chemical, as the areawide approach that is so strongly recommended as a foundational component of the *T. leucotreta* management programme only has the option of biological tools. Consequently, if a grower is in a region where SIT is not available, they have a choice of overlaying a biological approach (most likely virus sprays) or a chemical approach onto mating disruption. The average cost per hectare of a virus spray is 80 USD, whereas the average cost of a chemical spray is 243 USD. This is not considering the older chemistry, such as pyrethroids and some IGRs, whose use is discouraged or even restricted through residue intolerance by markets. Although the duration of control can be expected to be relatively similar between virus and chemical treatments, on average, it can be expected that the efficacy of a chemical treatment might be more consistent than that of a virus spray. However, the chemical treatments are more likely to have some detrimental effect on natural enemies, such as the parasitoids of *T. leucotreta* [46,55], thus eroding natural control, whereas this will not happen with the biological treatments. Consequently, there is little justification for preferring the chemical pesticides, particularly considering their higher cost.

Moore and Jukes [27] argue that the most compelling reason for using biopesticides and biological control agents is the argument of compulsion, rather than attraction. Tremendous pressure is being placed on the use of chemical insecticides [121,122]. Pesticide review programmes, increased stringency in the regulation of maximum residue levels (MRLs), arbitrary retailer residue restrictions, and growing public sentiment are contributing to this pressure [23]. The European Union (EU) has placed IPM centrally within its 2009 Sustainable Use Directive on pesticides [123]. EU member states were required to adopt national action plans for IPM by December 2012, and the implementation of these was reviewed in December 2018. This was most recently followed by the announcement of the European Green Deal, which aims to reduce net emission of greenhouse gasses in the region to zero by 2050 and by 50% by 2030 [124]. This will be done through substantial investment in environmentally friendly technologies. Similar directives and requirements might be expected for agricultural produce being imported into the EU, and other countries in the world may well follow the EU lead. Consequently, it is becoming more difficult for farmers to pursue a conventional chemical control programme, particularly if their produce is sold through high-end retailers or exported to OECD (Organisation for Economic Cooperation and Development) markets [27].

Consequently, in conclusion, it is not only possible to control the phytosanitary pest, *T. leucotreta*, for which export markets exercise zero tolerance, using exclusively biological means of control, but also highly desirable and beneficial for human (pesticide application) and environmental safety and in order to maintain the exportability of the fruit in a market that is increasingly chemical residue-intolerant.

## Figures and Tables

**Table 1:** Biopesticides developed or under development for control of *Thaumatotibia leucotreta* in citrus in South Africa.

Technology/Organism Category	Active Ingredient	Product Name	Commercial Company		Life Stage Targeted	Product Commercially Available
			Manufacturer	Local Supplier		
Sterile insect technique	Sterile male and female *T. leucotreta* adults	Xsit FCM SIT	Xsit	Xsit	Adult	Yes
Mating disruption	*T. leucotreta* female sex pheromone ^1,2^	Isomate FCM	Shin-Etsu	Nulandis	Adult male	Yes
		RB Splat FCM	ISCA Technologies	River Bioscience	Adult male	Yes
		Checkmate FCM-F	Suterra	Chempac	Adult male	Yes
		X-Mate FCM	Insect Science	Insect Science	Adult male	Yes
Attract and kill		Last Call FCM	Insect Science	Insect Science	Adult male	Yes
Parasitoids	*Trichogrammatoidea cryptophlebiae*	Tri-mX	Vital Bugs	Vital Bugs	Egg	Yes
Baculovirus	Cryptophlebia leucotreta granulovirus (CrleGV)	Cryptogran	River Bioscience	River Bioscience	Neonate larva	Yes
		Cryptex	Andermatt	Madumbi	Neonate larva	Yes
		Gratham	Andermatt	Chempac	Neonate larva	Yes
	Cryptophlebia peltastica nucleopolyhedrovirus (CrpeNPV)		River Bioscience	River Bioscience	Neonate larva	No
Entomopathogenic fungi	*Beauveria bassiana*	Broadband	BASF	BASF	Egg and neonate larva	Yes
		Eco-Bb	Plant Health Products	Madumbi	Egg and neonate larva	Yes
	Metarhizium anisopliae		River Bioscience	River Bioscience	Final instar	No
Entomopathogenic nematodes	*Heterorhabditis bacteriophora*	Cryptonem	e-nema	River Bioscience	All soil-dwelling life stages	Yes
	*Steinernema feltiae*	Nemapom	e-nema	River Bioscience	All soil-dwelling life stages	No

^1^ All products have two or three of the following isomers: (*E*)-7-dodecenyl acetate, (*E*)-8-dodecenyl acetate, (*Z*)-8-dodecenyl acetate, and (*E*/*Z*)-8-dodecenol. ^2^ Plus permethrin in the case of Last Call FCM.

**Table 2:** Cost comparison between biopesticides and chemical pesticides for control of *T. leucotreta* in citrus in South Africa. Prices were obtained from three local suppliers and averaged where necessary.

Biological or Chemical	Product ^1^	Cost per ha per Application or Programme ^2^ (USD)	Duration of Control per Application ^3^
Biological	Sterile insect technique (SIT)	309	6 months
	Isomate	287	6 months
	Splat	280	6 months
	Checkmate	323	6 months
	X-Mate	304	6 months
	Last Call	231	5 months
	Tri-mX	241	Season-long ^4^
	Cryptogran	81	4–17 weeks
	Cryptex	80	<Cryptogran ^5^
	Gratham	80	<Cryptogran ^5^
	Broadband ^6^	64	10 days
	Eco-Bb ^6^	62	10 days
Chemical	Delegate	73	4 weeks
	Runner	250	4 weeks
	Coragen	382	6–8 weeks
	Warlock	200	7–10 days

^1^ See Table 1 for product details. ^2^ This is the cost of a single application, except in the case of the mating disruption products and the attract-and-kill product, which are registered to be applied as a programme, consisting of more than one application. ^3^ Duration of control was obtained from the product label, except in the case of the virus products, which were obtained from Moore et al. [77]. ^4^ Difficult to give a duration of control, as augmentation is inoculative and, consequently, it will take some time for parasitoids to build up to an influential level. ^5^ Duration of residual efficacy is deemed to be shorter with these two virus products, as their amount of active ingredient per h is 7.6 × less than that of Cryptogran. ^6^ Dosage, duration of efficacy, and number of applications are not very clearly defined in the product recommendations on the label. Consequently, cost could be higher than listed.

## Data Availability

Not applicable.
